# A review of the pharmacological mechanism of corosolic acid

**DOI:** 10.3389/fphar.2026.1723882

**Published:** 2026-03-18

**Authors:** Xiaoyin Xu, Zhenduo Zhao, Jiaqi Zhang, Liang Shi, Wenhui Gao, Xi Yang, Xiaoting Zhang, Long Liu, Yanfeng Xu

**Affiliations:** 1 Department of Pharmacy, Shanghai Municipal Hospital of Traditional Chinese Medicine, Shanghai University of Traditional Chinese Medicine, Shanghai, China; 2 Tongji University Affiliated Tianyou Hospital, Shanghai, China

**Keywords:** AMPK, anti-cancer, anti-diabetes, corosolic acid, critical review, mechanism of action

## Abstract

Corosolic Acid (CA), a pentacyclic triterpenoid found in plants like Lagerstroemia speciosa, exhibits a wide array of preclinical pharmacological activities, including hypoglycemic, anti-cancer, anti-inflammatory, antioxidant, and cardiovascular protective effects. Often dubbed “plant insulin,” its therapeutic potential has garnered significant interest. However, despite numerous *in vitro* and animal studies elucidating potential mechanisms involving pathways like AMPK, NF-κB, YAP, and various kinases, a critical gap exists between this promising preclinical data and robust clinical validation. This review critically evaluates the existing evidence for CA’s major pharmacological actions, focusing on the strength and limitations of current mechanistic understandings, identifying key inconsistencies and controversies in the literature (searched via PubMed, Scopus, Cochrane, Web of Science, etc.). We particularly scrutinize the evidence supporting its roles in metabolic regulation and cancer therapy, highlighting areas where mechanistic insights remain superficial or conflicting. By synthesizing these findings, we aim to identify critical knowledge gaps and propose a structured roadmap with specific, hypothesis-driven future research directions required to rigorously assess CA’s translational potential and pave the way for potential clinical applications.

## 1 Introduction

Corosolic Acid (CA), a naturally occurring ursane-type pentacyclic triterpenoid (C_30_H_48_O_4_) with a molecular weight of 472.70 g/mol, is also known as 2α, 3β-dihydroxy-urs-12-en-28-oid acid. It can exist in free form or as saponins in plants and is extracted from various botanical sources, notably Banaba (*Lagerstroemia speciosa* L.) leaves (Magiera et al., 2021; Lourenço et al., 2021; Ulbricht et al., 2007), as well as *Prunus armeniaca* leaves (Móricz and Ott, 2022), *Prunus padus Linne* (Magiera et al., 2021), *Eucalyptus globulus* wood (Lourenço et al., 2021), and Chinese hawthorn (*Crataegus pinnatifida var.* major) leaves and so on. Table 1 summarizes the plants from which CA can be extracted. Its rigid, hydrophobic structure results in low aqueous solubility, prompting research into modified formulations, such as the addition of water-soluble groups like sugar or amino groups, to enhance its solubility (Qian et al., 2021). Since its initial association with glucose metabolism, earning it the moniker “plant insulin” (Stohs et al., 2012), preclinical research has burgeoned, suggesting a pleiotropic pharmacological profile encompassing anti-inflammatory (Gautam et al., 2015; Balakrishnan and Al-Assaf, 2016), antioxidant (Sinelius et al., 2023), anti-cancer (Xu et al., 2009; Peng et al., 2022; Zhang B. Y. et al., 2021), anti-viral (Vardhan and Sahoo, 2020), and cardiovascular protective effects (Wang et al., 2020; Luna-et al., 2018). Proposed mechanisms involve modulation of key cellular signaling pathways, including α-glucosidase inhibition (Feng et al., 2014; Zhang et al., 2017), AMP-activated protein kinase (AMPK) activation (Yang et al., 2016a; Liu et al., 2021; Wang et al., 2019), nuclear factor kappa B (NF-κB) inhibition (Gautam et al., 2015; Balakrishnan and Al-Assaf, 2016; Zhang et al., 2020), Yes-associated protein (YAP) pathway regulation (Xu et al., 2017; Jia et al., 2020; Zhang C. et al., 2021), and interference with growth factor receptor signaling (e.g., vascular endothelial growth factor receptor 2 (VEGFR2), human epidermal growth factor receptor 2/3 (HER2/3)) (Zhang B. Y. et al., 2021; Chang et al., 2015; Lee et al., 2010a; Li et al., 2019).

**TABLE 1:** Plants from which CA can be extracted.

Plant	Family	References
*Prunus armeniaca*. L	Rosaceae	Móricz and Ott (2022)
*Prunus padus* L	Rosaceae	Magiera et al. (2019)
*Eucalyptus globulus* Labill	Myrtaceae	Lourenço et al. (2021)
*Crataegus pinnatifida* var. *Major* N.E.Br	Rosaceae	Duan et al. (2021)
*Eriobotrya japonica* (Thunb.) Lindl	Rosaceae	Bao et al. (2021)
*Syzygium antisepticum (Blume)* Merr. and L.M. Perry	Myrtaceae	Mangmool et al. (2021)
*Salvia rosmarinus* Spenn	Lamiaceae	Sharma et al. (2020)
*Saurauia roxburghii* Wall	Actinidiaceae	Nahrin et al. (2020)
*Actinidia chinensis* Planch	Actinidiaceae	Fangchao (2020)
*Terminalia chebula* Retz	Combretaceae	Song et al. (2024)
*Solanum torvum* Sw	Solanaceae	Petchimuthu et al. (2023)
*Osmanthus fragrans *var. *Aurantiacus* (Makino) P.S.Green	Oleaceae	Han et al. (2024)
*Lagerstroemia speciosa* (L.) Pers	Lythraceae	Ulbricht et al. (2007)
*Potentilla chinensis* Ser	Rosaceae	Tran et al. (2025)
*Paulownia fortunei* (Seem.) Hemsl	Paulowniaceae	Lu et al. (2025)
*Psidium guajava* L	Myrtaceae	Srivastava et al. (2025)
*Angiopteris helferiana *C.Presl	Marattiaceae	Pandey et al. (2024)
*Cyclocarya paliurus* (Batalin) Iljinsk	Juglandaceae	Chen et al. (2024a)
*Crossopteryx febrifuga* (G.Don) Benth	Rubiaceae	Tchetan et al. (2024)
*Vaccinium macrocarpon *Aiton	Ericaceae	Xue et al. (2024)
*Punica granatum* L	Lythraceae	Chen et al. (2024b)
*Agrimonia pilosa* Ledeb	Rosaceae	Jiang et al. (2023)
*Miconia albicans* (Sw.) Steud	Melastomataceae	de Jesus et al. (2023)
*Glechoma hederacea* L	Lamiaceae	El-Aasr et al. (2023)
*Prunus mume* (Siebold) Siebold and Zucc	Rosaceae	Zhu et al. (2022)
*Handroanthus guayacan* (Seem.) S.O.Grose	Bignoniaceae	El-Hawary et al. (2022)
*Ancistrocarpus densispinosus* Oliv	Malvaceae	Peyeino et al. (2022)
*Chaenomeles speciosa* (Sweet) Nakai	Rosaceae	Lyu et al. (2021)
*Melaleuca citrina *(Curtis) Dum.Cours	Myrtaceae	Abdelmalek et al. (2021)
*Costus pictus* D. Don	Costaceae	Ansari et al. (2021)
*Eugenia pruniformis* Cambess	Myrtaceae	Albuquerque et al. (2020)
*Crataegus gracilior*	Rosaceae	Torres-Ortiz et al. (2020)
*Phyllanthus amarus* Schumach. and Thonn	Phyllanthaceae	Nhu et al. (2020)
*Avicennia marina* (Forssk.) Vierh	Acanthaceae	Nakamura et al. (2018)
*Scabiosa prolifera* L	Caprifoliaceae	Al-Qudah et al. (2017)
*Weigela subsessilis *(Nakai) L.H.Bailey	Caprifoliaceae	Feng et al. (2017)
*Plinia edulis* (Vell.) Sobral	Myrtaceae	Azevedo et al. (2016)
*Rubus stans* Focke	Rosaceae	Liu et al. (2016)
*Prunella vulgaris* L	Labiatae	Yang et al. (2016b)
*Malus domestica *(Suckow) Borkh	Rosaceae	Jemmali et al. (2016)
*Hippophaë rhamnoides *L	Elaeagnaceae	Jemmali et al. (2016)
*Salvia Sclarea* L	Lamiaceae	Jemmali et al. (2016)
*Perilla frutescens* (L.) Britton	Lamiaceae	Cho et al. (2015)
*Euphorbia lunulata* Bunge	Euphorbiaceae	Liu et al. (2015)
*Rosa cymosa* Tratt	Rosaceae	Huang et al. (2014)
*Potentilla discolor *Bunge	Rosaceae	Li et al. (2014)
*Psiloxylon mauritianum* (PM) (Bouton ex Hook.f.) Baill	Myrtaceae	Mahomoodally et al. (2014)
*Corymbia citriodora (Hook.)* K.D.Hill and L.A.S.Johnson	Myrtaceae	Wang et al. (2014)
*Vitex negundo* L	Verbenaceae	Chen et al. (2014)
*Potentilla fulgens* Wall. Ex Hook	Rosaceae	Choudhary et al. (2013)
*Lythrum salicaria* L	Lythraceae	Manayi et al. (2013)
*Clematoclethra scandens* (Franch.) Maxim. Subsp. *Actinidioides* Y. C. Tang et Q. Y. Xiong	Actinidiaceae	Xiao et al. (2013)
*Isodon japonicus* var. *Glaucocalyx* (Maxim.) H.W.Li	Lamiaceae	Yao et al. (2013)
*Eucommia ulmoides *Oliv. Leaf	Eucommiaceae	Li et al. (2012)
*Sambucus javanica *Reinw. Ex Blume	Adoxaceae	Tao and Fang (2012)
*Exochorda racemosa* (Lindl.) Rehder	Rosaceae	Zhang et al. (2011)
*Phlomis umbrosa* Turcz	Lamiaceae	Liu et al. (2009)
*Rubus biflorus *Buch.-Ham. ex Sm	Rosaceae	Kang et al. (2008)
*Platostoma africanum* P. Beauv	Lamiaceae	Aladedunye et al. (2008)
*Serissa japonica* (Thunb.) Thunb	Rubiaceae	Li et al. (2007)
*Glechoma longituba *(Nakai) Kuprian	Lamiaceae	Yang et al. (2006)

Despite this wealth of preclinical data, the clinical translation of CA remains notably limited. Unlike previous systematic reviews, this review critically analyzes CA research, revealing underlying flaws: Overreliance on *in vitro* studies and animal models, with insufficient human clinical trials; inadequate research on the pharmacokinetics (PK), pharmacodynamics (PD), and active metabolites of CA; inconsistent and contradictory findings across various studies on mechanisms and efficacy.

This review adopts a critical perspective, we move beyond listing biological activities to critically examine core mechanistic debates (e.g., the primacy of AMPK activation vs. direct enzyme inhibition), apparent paradoxes (e.g., context-dependent effects on ROS), and cross-talk between pathways. We will dissect key controversies, highlight knowledge gaps, particularly concerning the interplay between its diverse cellular targets, and propose a forward-looking research strategy essential for bridging the significant translational gap between benchtop observations and potential bedside applications.

## 2 Methods (literature search strategy)

This critical narrative review was conducted to comprehensively evaluate the multifaceted pharmacological profile of corosolic acid. A systematic literature search was performed across multiple academic databases (PubMed, Scopus, Cochrane Library, ScienceDirect, and Web of Science) up to March 2025. The search strategy employed a combination of key terms, including “Corosolic Acid”, “pharmacology”, “mechanism”, “diabetes”, “cancer”, “inflammation”, “cardiovascular”, “AMPK”, “NF-κB”, “YAP”, and related MeSH/Emtree terms.

The search was designed to identify primary research articles and seminal reviews reporting on the mechanistic insights, *in vitro* and *in vivo* efficacy, and formulation development of CA. Study selection prioritized publications with clearly described methodologies and quantitative data relevant to CA’s biological effects and underlying mechanisms. Identified studies were then critically appraised for their evidence strength, limitations, internal consistency, and translational relevance.

**FIGURE 1:**
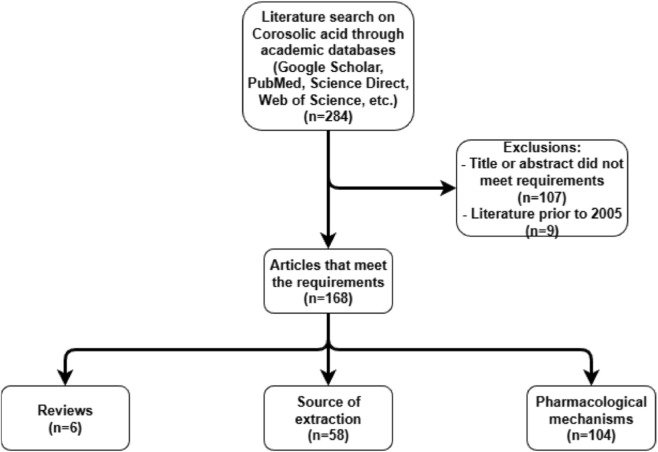
Search and selection overview of the articles of CA.

While earlier reviews have often detailed CA’s action within a single disease context, this review synthesizes evidence across metabolic, oncological, and inflammatory domains. It aims to provide an integrated analysis of CA’s major pharmacological actions and the complex network of signaling pathways involved, thereby identifying current knowledge gaps and laying a groundwork for future translational research (Figure 1).

## 3 Results and critical evaluation of evidence

### 3.1 Metabolic regulation: hypoglycemic and hypolipidemic effects

CA has demonstrated significant potential in metabolic regulation, particularly exhibiting anti-diabetic activity in various cell and animal models, with some preliminary evidence in humans (Ulbricht et al., 2007; Stohs et al., 2012). Its hypoglycemic and insulin-sensitizing effects are attributed to several complementary mechanisms. These include the enhancement of insulin signaling and glucose uptake, partly mediated through the activation of AMPK (Yang et al., 2016a) and potential inhibition of negative regulators of insulin signaling such as protein-tyrosine phosphatase 1B (PTP1B). CA may also exert potential insulin-mimetic activity (Stohs et al., 2012). Another key mechanism involves the inhibition of carbohydrate-digesting enzymes, specifically through the non-competitive inhibition of α-glucosidase (Feng et al., 2014; Zhang et al., 2017; Liu et al., 2020; Ni et al., 2019), which reduces postprandial glucose absorption. Furthermore, CA contributes to metabolic health by reducing inflammation and oxidative stress in metabolic tissues (Yang et al., 2016a). This is achieved through the downregulation of pro-inflammatory pathways like NF-κB (Gautam et al., 2015; Balakrishnan and Al-Assaf, 2016; Zhang et al., 2020) and stress-activated kinases such as p38 mitogen-activated protein kinase (MAPK)/extracellular signal-regulated kinase (ERK)1/2 (Li X-Q. et al., 2016) and c-Jun N-terminal kinase (JNK), leading to improved insulin sensitivity. Beyond glucose metabolism, CA has shown promise in mitigating diabetic complications, including nephropathy *via* inhibiting p38 MAPK/ERK1/2 signaling (Li X-Q. et al., 2016) and potentially retinopathy through anti-angiogenic effects (Toledo et al., 2019). It also inhibits aldose reductase and the formation of advanced glycation endproducts (AGEs) (Rao et al., 2012). Lipid-lowering effects have also been suggested, potentially through the inhibition of cholesterol uptake and acyl-CoA:cholesterol acyltransferase-2 (ACAT-2) (Takagi et al., 2010), as well as the regulation of hepatic lipogenesis via AMPK/sterol regulatory element-binding proteins (SREBPs) and NF-κB/MAPK pathways, particularly demonstrated in models of non-alcoholic fatty liver disease (NAFLD) and non-alcoholic steatohepatitis (NASH) (Liu et al., 2021; Zhang et al., 2020). Protective effects against alcoholic liver disease (ALD) have also been linked to CA, potentially involving the inhibition of store-operated calcium entry (SOCE) and modulation of MAPK/autophagy pathways (Guo et al., 2016; Cui et al., 2015). These diverse mechanisms have been elucidated through *in vitro* experiments (e.g., using adipocytes, muscle cells, enzyme assays) and validated in various *in vivo* studies, primarily using diabetic rodent models (Miura et al., 2006; Shi et al., 2008).

Delving deeper into the molecular mechanisms, a key anti-diabetic action of CA is the improvement of insulin sensitivity in peripheral tissues. In diabetic mouse models like the KK-A^y strain, CA supplementation (e.g., 2 mg/kg) resulted in significantly lower fasting blood glucose and insulin levels after 2 weeks, alongside enhanced glucose clearance in insulin tolerance tests, indicating improved insulin action (Miura et al., 2006). At the molecular level, CA enhances insulin receptor (IR) signaling in skeletal muscle. Studies using L6 myotubes and other insulin-responsive cells demonstrate that CA increases IR phosphorylation and downstream Protein kinase B (Akt) activation, consequently promoting glucose transporter type 4 (GLUT4) translocation to the cell membrane (Shi et al., 2008). These insulin-mimetic effects are potent enough to be blocked by the Phosphoinositide 3-kinase (PI3K) inhibitor wortmannin, confirming the involvement of the canonical insulin signaling pathway. One proposed mechanism for this enhancement is CA’s inhibition of protein-tyrosine phosphatases like PTP1B, which normally attenuate insulin signaling; by inhibiting PTP1B, CA sustains IR phosphorylation, thereby potentiating insulin action. Concurrently, CA activates AMPK, an energy-sensing kinase that improves metabolic parameters independently of insulin. In obese high-fat diet (HFD)-fed mice, chronic CA treatment improved insulin sensitivity and glucose tolerance, effects strongly linked to AMPK activation in metabolic tissues such as adipose tissue (Yang et al., 2016a). Importantly, knockdown of AMPKα in 3T3-L1 adipocytes or pharmacological inhibition of AMPK abolished CA’s anti-inflammatory and insulin-sensitizing effects, indicating that CA acts through an AMPK-dependent mechanism to alleviate insulin resistance (Yang et al., 2016a). Activated AMPK enhances glucose uptake in muscle and suppresses hepatic glucose production, contributing to the observed glycemic control. Indeed, enhanced insulin signal transduction (e.g., higher Akt phosphorylation) was noted alongside AMPK activation in CA-treated HFD mice. Thus, CA appears to engage both insulin-dependent pathways (by amplifying IR/Akt signaling) and insulin-independent pathways (via AMPK activation) to promote glucose utilization.

In addition to improving insulin responsiveness, CA may mitigate postprandial hyperglycemia by reducing carbohydrate absorption in the gut through the inhibition of α-glucosidase (Feng et al., 2014; Zhang et al., 2017; Liu et al., 2020), a key intestinal enzyme. Enzyme kinetics assays revealed that CA inhibited yeast α-glucosidase in an uncompetitive manner, with an IC50 value on the order of 10–15 μM (Ni et al., 2019). Molecular docking studies suggest CA binds near the active site, hindering substrate processing (Liu et al., 2020). This *in vitro* potency is comparable to some pharmaceutical α-glucosidase inhibitors (Zhang et al., 2017), implying that CA can slow glucose release from dietary carbohydrates. Supporting this, co-administration of CA has been found to blunt postprandial blood glucose spikes in animal studies and humans. A small clinical study involving 31 subjects showed that a single 10 mg oral dose of CA given 5 min before a 75 g oral glucose tolerance test resulted in lower plasma glucose levels at 60–120 min (significant at 90 min) compared to placebo. Similarly, a trial reported in 2006 found that CA supplementation before a carbohydrate challenge significantly reduced 2-h postchallenge glucose levels in humans versus controls (Fukushima et al., 2006). These findings, although indicating a moderate glucose-lowering effect [a reduction on the order of 10%–15% in postprandial levels (Stohs et al., 2012)], align with an α-glucosidase inhibitory effect *in vivo*. Notably, the effective dose in humans (10 mg) is relatively low, suggesting CA or Banaba extract has efficacy at nutraceutical dosing levels.

Chronic low-grade inflammation in adipose and liver tissue is a known contributor to insulin resistance in type 2 diabetes, and CA appears to counteract this inflammatory milieu. In HFD-fed mice, CA treatment suppressed NF-κB activation in adipose tissue, evidenced by reduced phosphorylation of inhibitor of nuclear factor kappa-B kinase subunit beta (IKKβ) (the kinase activating NF-κB) and lowered gene expression of proinflammatory cytokines like tumor necrosis factor alpha (TNFα) and interleukin-6 (IL-6) (Yang et al., 2016a). Histologically, CA-treated obese mice exhibited less macrophage infiltration in adipose tissue, indicating alleviation of adipose inflammation (Yang et al., 2016a). By preventing NF-κB-mediated inflammatory signaling (Gautam et al., 2015; Balakrishnan and Al-Assaf, 2016; Zhang et al., 2020), CA helps preserve IR sensitivity, as inflammation often induces inhibitory serine phosphorylation of insulin receptor substrate 1 (IRS-1). Consistent with this, CA-treated mice showed reduced serine phosphorylation of IRS-1, correlating with improved downstream Akt activation. There is also evidence that CA can inhibit the stress-activated kinases JNK and p38 MAPK (Li X-Q. et al., 2016), which are typically elevated in obesity and interfere with insulin action. In a rat model of metabolic syndrome, CA administration prevented oxidative stress and lowered the activation of JNK/p38 pathways in tissues, accompanied by reduced inflammatory markers and associated with improved insulin sensitivity and even blood pressure reduction (Yamaguchi et al., 2006), highlighting its broad metabolic benefits. Mechanistically, part of this effect may stem from CA’s ability to reduce reactive oxygen species (ROS) generation in metabolic tissues, thereby breaking a key link in the inflammation-insulin resistance cycle. Overall, by quelling inflammation (via NF-κB, JNK, p38 modulation) and potentially improving adipokine profiles, CA helps restore normal insulin signaling in insulin-resistant states.

The anti-diabetic mechanisms of CA have been demonstrated across multiple experimental systems. *In vitro*, aside from enzyme assays (α-glucosidase) (Feng et al., 2014; Zhang et al., 2017; Liu et al., 2020) and cell culture studies (hepatocytes, myotubes, adipocytes), reports indicate CA directly stimulates glucose uptake in muscle cells. For example, CA increased glucose uptake in L6 muscle cells by approximately 30%–40% compared to baseline, an effect comparable to insulin in some cases. Such findings underpin the biochemical basis for CA’s reputation as a “phyto-insulin” (Stohs et al., 2012). *In vivo*, multiple rodent studies validate that CA can improve glycemic control. Diabetic KK-Ay mice treated with CA showed significant drops in blood glucose both acutely (within 4 h of a single dose) and chronically (lower glucose levels after 2 weeks of daily dosing) (Miura et al., 2006). Importantly, their plasma insulin was also lower, indicating improved insulin sensitivity rather than simply stimulating insulin secretion. CA has also been tested in obese hypertensive rats (SHR/cp), where it reduced fasting glucose and insulin, and improved the insulin tolerance test response relative to controls, consistent with enhanced whole-body insulin sensitivity. While human data remain limited, the aforementioned small trials with CA or Banaba leaf extract indicate a tangible, if moderate, glucose-lowering effect in the context of a glucose challenge (Shi et al., 2008; Fukushima et al., 2006). One study using a *Lagerstroemia speciosa* (Banaba) extract reported a dose-dependent decrease in blood glucose in type II diabetic patients over several weeks, suggesting CA’s effects may extend beyond acute postprandial changes (Stohs et al., 2012). However, large-scale clinical trials are still lacking.

A critical evaluation of CA’s anti-diabetic potential must address its PK limitations. CA is a pentacyclic triterpenoid characterized by very low water solubility (Qian et al., 2021), which leads to poor oral absorption (Agarwal et al., 2018). Consequently, only a small fraction of orally administered CA is absorbed into the bloodstream, and it tends to exhibit a short plasma half-life. These factors might explain why relatively high doses of CA are often required in studies to observe effects, and why dietary supplements containing banaba necessitate consistent intake. Some studies explicitly note these issues, highlighting CA’s “poor pharmacokinetic properties” and even potential cell toxicity at higher concentrations (Huang et al., 2025). To overcome this significant barrier, researchers have explored various formulation strategies. For instance, a self-microemulsifying drug delivery system (SMEDDS) for banaba extract (standardized to CA content) was shown to dramatically improve CA’s absorption and efficacy (Agarwal et al., 2018). In diabetic rats, this emulsion-based formulation produced a greater reduction in blood glucose at a 50 mg/kg dose compared to the same dose of plain extract, and enabled a significant glucose-lowering effect at half the dose (25 mg/kg) required for the plain extract (Agarwal et al., 2018). This suggests that enhancing solubility and dissolution can markedly improve CA’s bioavailability and therapeutic potential. Despite these advances, it is important to note that many preclinical studies delivered CA via routes like intraperitoneal injection or dissolved in organic solvents for gavage, methods not directly applicable to human oral use. Therefore, while CA possesses intrinsic antidiabetic activity, a key challenge moving forward is developing formulations optimized for human oral delivery. Encouragingly, the moderate doses effective in rodents (e.g., 2–10 mg/kg) might translate to a manageable range for humans (potentially around 100 mg daily, based on body surface area scaling), especially if advanced formulation technologies are employed.

Critically evaluating the evidence reveals several considerations and controversies. Regarding evidence strength, while α-glucosidase inhibition is demonstrated *in vitro* (Feng et al., 2014; Zhang et al., 2017; Liu et al., 2020), its *in vivo* contribution to overall glucose lowering compared to AMPK activation (Yang et al., 2016a) or other effects remains unclear. The “plant insulin” claim (Stohs et al., 2012), stemming from its insulin-mimetic activities, lacks robust mechanistic validation beyond initial observations. Evidence for AMPK activation appears more consistent (Yang et al., 2016a; Liu et al., 2021), though dose-dependency and isoform specificity require further characterization. Anti-angiogenic effects reported for potential retinopathy treatment (Toledo et al., 2019) are based on *in vitro* (CAM assay) and *in vitro* data, lacking *in vivo* validation and PK data for ophthalmic delivery. Limitations include the reliance of many studies on HFD or chemically-induced animal models (Liu et al., 2021; Zhang et al., 2020; Alkholifi et al., 2023) (e.g., CCl_4_, STZ), whose pathologies may not fully mirror complex human metabolic diseases like T2D, NAFLD, or ALD. The clinical relevance of inhibiting specific enzymes like ACAT-2 *in vivo* requires confirmation (Takagi et al., 2010). The low water solubility and consequent poor bioavailability remain significant hurdles (Qian et al., 2021). Key controversies and research gaps persist: What is the primary hypoglycemic mechanism of CA *in vivo* - direct enzyme inhibition, AMPK-mediated sensitivity enhancement, or anti-inflammatory action? How significant is its lipid-lowering effect compared to standard therapies? The specific molecular interactions underpinning the inhibition of pathways like p38/ERK (Li X-Q. et al., 2016) or SOCE (Guo et al., 2016; Cui et al., 2015) need deeper investigation. The potential synergy with existing drugs like acarbose (Zhang et al., 2017) or metformin (both impacting AMPK) warrants rigorous exploration. Furthermore, dose-dependence and potency need clarification; while micromolar concentrations are effective *in vitro*, whether these are achieved and sustained in target tissues at typical human supplement doses (e.g., 10 mg) to exert significant effects (like direct PTP1B or α-glucosidase inhibition) is questionable, necessitating more PK-PD correlation data. CA’s polypharmacology, influencing numerous pathways, while potentially beneficial for multifactorial conditions, also raises concerns about off-target effects (e.g., ubiquitous PTP1B inhibition), although severe issues have not been reported in animal studies. Significant gaps exist in clinical evidence, particularly regarding long-term glycemic control (glycated hemoglobin A1c (HbA1c) reduction) and safety in diabetic patients. It remains unclear whether CA primarily acts as a mild nutraceutical supplement or could be developed into a potent antidiabetic drug. Finally, the context-dependent effects on oxidative stress are notable: while CA generally exhibits beneficial antioxidant activity in metabolic tissues (reducing ROS and inflammation), it can promote ROS generation in cancer cells, highlighting the importance of dosage and cellular context. The doses used in diabetic models appear to alleviate oxidative stress (Tidke and Patil, 2017), suggesting a favorable safety margin in this context, but underscoring the need for careful dose evaluation in any future human use. Table 2 summarizes the antiglycemic and hypolipidemic mechanisms of CA.

**TABLE 2:** Hypoglycemic and hypolipidemic mechanism of CA (*In vitro* and *vivo*).

Diseases	Cell/Animals	Doses	Mechanisms	Bibliography
*In vitro* models				
T2D	Glomerular mesangial cells ERK 1/2 and p38 MAPK	0.3–300 μM 30 μM	Introduced a potent α-glucosidase inhibitor at the C-28 position of and CA by esterification and nucleophilic substitution reaction	Li et al. (2016a)
Diabetic nephropathy	MAPK; NADPH; RK1/2	10 μM	Inhibitded and inactivated p38 MAPK and NADPH-mediated e ERK1/2	Yang et al. (2016a)
Insulin resistance	IKKβ; AMPK	9.79 μM	Inhibited IKKβ phosphorylation, downregulating the expression of pro-inflammatory cytokines activated AMPK, attenuated inflammatory responses in adipose tissue	Rao et al. (2012)
Retinal neovascularization	ARPE-19	5–25 μM	Initial CA concentrations of 5–25 μM induced a significant reduction in vascular distribution without signs of toxicity	Toledo et al. (2019)
NAFLD	AMPK/SREBPs and NF-κB/MAPK signaling pathways	0, 5, 10, 20 μM	Exerted effects through AMPK/SREBPs and NF-κB/MAPK signaling pathways, ultimately inhibited hepatic lipogenesis, cholesterol synthesis, and inflammation	Zhang et al. (2020)
*In vivo* models				
T2D	High fat diet-fed mice	0.017%w/w	Could reverse the pathological process of T2DM through regulating the disturbed pathways of metabolism including purine metabolism, amino acid metabolism, tryptophan metabolism and lipid metabolism	Feng et al. (2016)
	STZ-induced diabetic rats Diabetic db/db mice	60 mg/kg 10 mg/kg	Introduced a potent α-glucosidase inhibitor at the C-28 position of and CA by esterification and nucleophilic substitution reaction	Li et al. (2016a)
Diabetic nephropathy	High fat diet-fed mice	20 mg/kg	Improved glucose tolerance and alleviated inflammation	Yang et al. (2016a)
Long-term diabetic	Diabetic mice	10 mg/kg, i.g	Inhibited AR activity in rat lens, rat kidney, and human recombinant cells	Papatheodorou et al. (2018)
NAFLD	NAFLD model mice	15, 30 mg/kg	Exerted effects through AMPK/SREBPs and NF-κB/MAPK signaling pathways, ultimately inhibited hepatic lipogenesis, cholesterol synthesis, and inflammation	Zhang et al. (2020)
ALD	ALD model rats	20% CA 4 mL, i.g	Inhibited store-operated calcium entry (SOCE) triggered by intracellular calcium stores	Cui et al. (2015)
	ALD model rats	20% CA 4 mL, i.g. Twice a day	Prevented ethanol-induced liver injury by regulating MAPK signaling and autophagy activation	Guo et al. (2016)
liver toxicity	Rat model of hepatotoxicity induced by INH and DDS	500 mg/kg (CA contained in ethanolic banaba leaves extract)	Maintenance of liver cell membrane stability	Rohit Singh and Ezhilarasan (2021)
	CCL4-induced-hepatotoxic rats	20 mg/kg	Inhibited TNF-α, IL-6, iNOS, COX-2, and NF-κB, observed both *in vitro* and *in vivo*, decreased their mRNA expression levels	Balakrishnan and Al-Assaf (2016)

### 3.2 Anti-cancer effects

Beyond its metabolic benefits, CA has exhibited broad-spectrum anti-tumor effects across numerous cancer types *in vitro* and in some xenograft models. These include hepatocellular carcinoma (HCC), cholangiocarcinoma (CCA), gastric, colorectal (CRC), pancreatic, lung, bladder, renal, prostate, breast, ovarian, skin, retinoblastoma (RB), oral, osteosarcoma, glioblastoma (GBM), and leukemia (Wirsching et al., 2016; Fujiwara et al., 2011; Uto et al., 2013). These anti-cancer properties are manifested through multiple, often overlapping, mechanisms. Key reported actions include the induction of cancer cell apoptosis (Xu et al., 2009; Uto et al., 2013; Jedram et al., 2023; Lee et al., 2010b; Cheng et al., 2017; Sung et al., 2014; Nho et al., 2013; Ma et al., 2018; Jasim et al., 2023; Cai et al., 2011; Jia et al., 2016), induction of cell cycle arrest (Lee et al., 2010a; Cui et al., 2023; Wang et al., 2018), and the inhibition of critical tumor growth and survival signaling pathways. Notable pathways inhibited by CA include the Hippo/YAP pathway (Xu et al., 2017; Jia et al., 2020; Zhang C. et al., 2021), Janus kinase (JAK)/signal transducer and activator of transcription 3 (STAT3) signaling (Cho et al., 2015; Fujiwara et al., 2011; Jasim et al., 2023; Fujiwara et al., 2013; Horlad et al., 2013), mammalian target of rapamycin (mTOR) signaling (Lee et al., 2010b; Lee et al., 2015), HER2/HER3 receptor tyrosine kinase activity (Zhang B. Y. et al., 2021; Lee et al., 2010a), VEGFR2 signaling (Chang et al., 2015; Li et al., 2019), the Wnt/β-catenin pathway (Kim et al., 2014), focal adhesion kinase (FAK)/ERK signaling (Yoo et al., 2015), PI3K/AKT pathways (Ma et al., 2018; Jin et al., 2021), and the AXL receptor tyrosine kinase (AXL) axis (Sun et al., 2021). Additionally, CA can induce oxidative stress in tumor cells, leading to ROS-dependent apoptosis (Nho et al., 2013) or ferroptosis-like cell death (Peng et al., 2022; Jin et al., 2024; Woo et al., 2018). CA also interferes with the tumor microenvironment by reducing pro-tumor inflammation (e.g., inhibiting cyclooxygenase-2 (COX2)/NF-κB (Han et al., 2024), suppressing M2 macrophage polarization (Fujiwara et al., 2011; Zhao et al., 2022)), inhibiting angiogenesis (Chang et al., 2015; Toledo et al., 2019; Yoo et al., 2015), and modulating immunosuppressive cells like myeloid-derived suppressor cells (MDSCs) (Horlad et al., 2013). Other reported mechanisms encompass the inhibition of YAP O-GlcNAcylation via cyclin-dependent kinase 19 (CDK19) (Zhang C. et al., 2021), induction of PKR-like endoplasmic reticulum kinase (PERK)-mediated endoplasmic reticulum (ER) stress (Ma et al., 2018; Tang F-F. et al., 2023; Tang F. et al., 2023) [targeting prolyl 4-hydroxylase subunit alpha 2 (P4HA2) (Tang F-F. et al., 2023)], disruption of microtubules (Li et al., 2019), attenuation of hypoxia-inducible factor 1-alpha (HIF-1α) (Bahadori et al., 2019), overcoming chemoresistance (e.g., to 5-fluorouracil (5-FU) via AMPK (Park et al., 2018), to cisplatin via targeting protein for Xklp2 (TPX2)/PI3K/AKT and ROS modulation (Jin et al., 2021), to chemotherapy in ovarian cancer via STAT3 (Fujiwara et al., 2013)), induction of mitophagy (Cui et al., 2023), inhibition of matrix metalloproteinases (MMPs) (Wu et al., 2023; Chen et al., 2021), activation of nuclear factor erythroid 2-related factor 2 (Nrf2) and epigenetic modulation (Yang et al., 2018; Hudlikar et al., 2020), inhibition of secretory phospholipase A2 group IIa (sPLA2IIa) (Pundalik et al., 2022), inhibition of epidermal growth factor receptor (EGFR) signaling (Cho et al., 2015), disruption of maternal embryonic leucine zipper kinase (MELK)-forkhead box M1 (FoxM1) signaling in RB (Wang et al., 2018), and induction of mitochondrial dysfunction (Uto et al., 2013). Both *in vitro* experiments on diverse cancer cell lines and *in vivo* tumor models (xenografts, metastasis models) have been employed to evaluate CA’s efficacy.

The induction of apoptotic cell death is one of the most consistently reported effects of CA on cancer cells. CA triggers apoptosis via both the intrinsic mitochondrial pathway [e.g., modulating BCL2 associated X, apoptosis regulator (Bax)/B-cell lymphoma 2 (Bcl-2) ratio (Jedram et al., 2023; Sung et al., 2014), activating caspases (Xu et al., 2009; Jedram et al., 2023; Sung et al., 2014; Cai et al., 2011)] and the extrinsic death-receptor pathway, depending on the cellular context. For instance, in human CRC HCT116 cells, CA treatment led to activation of caspases (−8, −9, and −3) and altered expression of pro- and anti-apoptotic proteins (Bax, Fas ligand (FasL) increased; Bcl-2, survivin decreased) (Sung et al., 2014). Caspase inhibitors could rescue cells, confirming caspase-dependent apoptosis. Similarly, in human gastric cancer cell lines (e.g., BGC-823), CA (10–80 μg/mL) induced dose-dependent apoptosis, potentially linked to inhibition of the NF-κB pathway (downregulation of p65 nuclear translocation) (Cheng et al., 2017). Apoptosis induction by CA has also been observed in hormone-related cancers; in castration-resistant prostate cancer cells (PC-3 and DU145), CA (10–50 µM) induced ER stress-mediated apoptosis, marked by upregulation of ER stress sensors (inositol-requiring enzyme 1 alpha (IRE1α), C/EBP homologous protein (CHOP)) and activation of the JNK pathway, culminating in caspase activation (Ma et al., 2018). This effect translated to growth inhibition in a mouse xenograft model of PC-3. Notably, CA’s apoptotic effects often occur alongside cell cycle arrest, for example, causing accumulation in the G0/G1 phase in HCT116 cells (Sung et al., 2014), potentially sensitizing cells to apoptotic triggers.

Emerging evidence indicates that CA can inhibit the activity of YAP, an oncogenic transcription co-activator frequently dysregulated in cancer *via* the Hippo pathway. In HCC, CA treatment in cell lines led to decreased nuclear localization of YAP and reduced expression of YAP-regulated target genes (e.g., connective tissue growth factor (CTGF)), thereby impeding HCC cell proliferation and migration (Jia et al., 2020). Mechanistically, CA may facilitate large tumor suppressor kinase 1 (LATS1)/beta-transducin repeat containing E3 ubiquitin protein ligase (β-TrCP)-mediated ubiquitination and degradation of YAP (Xu et al., 2017). Supporting YAP as a critical target, combining CA with actinomycin D (which can also suppress YAP transcriptional output) produced synergistic anti-tumor effects in liver cancer models, more effectively reducing HCC cell viability and tumor growth in mice than either agent alone (Xu et al., 2017; Zhang C. et al., 2021). This highlights YAP inactivation as a significant mechanism, particularly relevant for cancers like HCC. By inactivating YAP, CA may also curb migration, invasion, and potentially metastasis (Jia et al., 2020).

CA’s influence extends to the tumor microenvironment, notably through modulation of the JAK/STAT3 pathway. STAT3 is commonly activated in cancer cells and tumor-associated immune cells (like M2 macrophages and MDSCs), promoting tumor survival, proliferation, and immunosuppression. Studies demonstrate CA acts as a STAT3 inhibitor in various cancer contexts (Cho et al., 2015; Fujiwara et al., 2011; Jasim et al., 2023; Fujiwara et al., 2013; Horlad et al., 2013). In ovarian cancer, CA (at non-cytotoxic doses ∼20–30 µM) inhibited IL-6-induced STAT3 phosphorylation in epithelial cancer cells and suppressed the polarization of co-cultured macrophages toward the pro-tumor M2 phenotype (Fujiwara et al., 2013). Fujiwara et al. showed CA enhanced chemotherapy efficacy (paclitaxel, cisplatin, doxorubicin) on ovarian carcinoma cells by blocking STAT3 activation, reducing chemoresistance and tumor-supporting macrophages *in vitro* and in mouse models (Fujiwara et al., 2013). Similarly, in GBM, CA (around 30 µM) suppressed STAT3 (and NF-κB) activation in both glioma cells and M2-polarized microglia, correlating with reduced tumor cell proliferation (Fujiwara et al., 2011). The *in vivo* relevance of STAT3 modulation is exemplified by studies in tumor-bearing mice. Horlad et al. found that oral CA (17.5 mg/kg daily) dramatically impaired subcutaneous sarcoma growth and significantly reduced lung metastasis (Fujiwara et al., 2011). CA-treated tumors showed increased infiltration of CD4^+^ and CD8^+^ T lymphocytes alongside decreased MDSC immunosuppressive activity, mechanistically tied to CA’s suppression of STAT3 signaling in MDSCs and tumor cells (Horlad et al., 2013). This suggests CA might synergize with immunotherapies by remodeling the tumor immune microenvironment towards an anti-tumor response.

While CA often acts as an antioxidant in metabolic disease contexts, in cancer cells it frequently promotes oxidative stress. Treatment with CA elevates ROS levels in multiple cancer cell types (Nho et al., 2013; Jin et al., 2021). Lipid ROS (peroxidation of membrane lipids) appears critical for CA-induced death in some cancers. For example, in human renal carcinoma Caki cells, CA (∼10 µM) induced a non-apoptotic cell death characterized by a surge in lipid peroxides (Woo et al., 2018). This death was not prevented by classical ferroptosis inhibitors but was rescued by antioxidants targeting lipid ROS, such as vitamin E (α-tocopherol), suggesting CA triggers a lipid peroxidation-dependent, potentially ferroptosis-like, non-apoptotic death pathway (Woo et al., 2018). This capacity to engage alternative death pathways is advantageous against apoptosis-resistant cells. Notably, CA was less toxic to normal human mesangial cells at equivalent doses in the same study, suggesting preferential vulnerability of cancer cells (Woo et al., 2018). Further studies indicate CA can sensitize liver (Peng et al., 2022) and pancreatic (Jin et al., 2024) cancer cells to ferroptosis, potentially by upregulating homocysteine inducible ER protein with ubiquitin like domain 1 (HERPUD1) (Peng et al., 2022) or via spermidine/spermine N1-acetyltransferase 1 (SAT1)-mediated mechanisms (Jin et al., 2024). ROS generation by CA can also act upstream, triggering apoptosis (e.g., in A549 lung cancer cells (Nho et al., 2013)) or activating signaling cascades like JNK contributing to ER stress-induced apoptosis in prostate cancer (Ma et al., 2018).

CA’s broad anticancer effects involve interference with numerous other oncogenic signaling axes. It has been identified as a novel inhibitor of the HER2/HER3 heterodimer, blocking dimerization and downstream AKT/ERK signaling in HER2-positive cancers (Zhang B. Y. et al., 2021). It also promotes the degradation of the AXL receptor tyrosine kinase by enhancing CHIP-mediated ubiquitination, thereby inhibiting the growth arrest specific 6 (GAS6)/AXL/JAK/STAT pathway and reducing GBM cell invasiveness (Sun et al., 2021). In CRC models with adenomatous polyposis coli (APC) mutations, CA downregulates β-catenin, reduces Wnt target gene expression, and impairs proliferation (Kim et al., 2014). A recent study in liver cancer cells found CA reduces O-GlcNAcylation by downregulating CDK19, leading to decreased C activity and subsequent growth arrest/apoptosis (Zhang C. et al., 2021). Given this wide array of targets, CA likely exerts multi-targeted pressure on cancer cells, potentially thwarting resistance but also complicating mechanistic interpretation, as context (cancer type, genetics, microenvironment) likely determines the dominant pathway.

Tumor progression relies on angiogenesis and metastasis, processes that CA appears to impede. In a CRC CT-26 xenograft model, peritumoral CA administration (5 or 25 mg/kg/day) reduced tumor growth and microvessel density (Yoo et al., 2015). Treated tumors had lower expression of angiogenesis factors like angiopoietin-1, and reduced phosphorylation of FAK and ERK in endothelial cells (Yoo et al., 2015). *In vitro* assays confirm CA inhibits endothelial cell tube formation, partly by suppressing VEGF-driven pathways (Chang et al., 2015; Toledo et al., 2019). CA also reduced lymphangiogenesis in the tumor model (Yoo et al., 2015). Regarding metastasis, CA’s inhibition of YAP (Jia et al., 2020), STAT3 (Horlad et al., 2013), and MMPs (Wu et al., 2023; Chen et al., 2021) likely contributes to reduced invasion. The significant reduction in lung metastasis (85% fewer nodules) observed in the sarcoma model highlights CA’s potential to hamper cancer spread (Horlad et al., 2013), although more research across different cancer models is needed.

A recurring theme is CA’s synergy with standard cancer therapies. It reverses chemoresistance to 5-FU in gastric cancer cells by activating AMPK and suppressing mTOR (Lee et al., 2015; Park et al., 2018). In ovarian cancer, CA enhances the apoptotic effects of chemotherapy (Fujiwara et al., 2013). Its ability to reduce MDSCs and promote T cell infiltration suggests synergy with immune checkpoint inhibitors (Horlad et al., 2013). Combination with targeted therapy, like sorafenib in HCC mouse xenografts, also showed enhanced tumor growth suppression without added toxicity (Chang et al., 2015). These findings support integrating CA into multi-modal treatment regimens.

While *in vitro* data are abundant, *in vivo* evidence provides crucial validation. Several studies using mouse models (human cancer xenografts, syngeneic models) have demonstrated CA’s anti-tumor activity at doses ranging roughly from 5 to 50 mg/kg (Zhang B. Y. et al., 2021; Chang et al., 2015; Ma et al., 2018; Cui et al., 2023; Horlad et al., 2013; Luo et al., 2024). Reported tumor volume reductions range from 50% to 85% compared to controls, often without overt toxicity at effective doses. However, the administration routes used (e.g., intraperitoneal, peritumoral injection) may achieve higher local concentrations than feasible via oral administration in humans. To date, no human clinical trial data for CA in cancer have been published. Therefore, its relevance to human disease is inferred from preclinical results and mechanistic understanding. While actions on conserved cancer pathways suggest broad applicability, the existence of these pathways in normal cells raises potential toxicity concerns (e.g., impacting normal STAT3 or YAP functions), although animal studies have not reported major issues. Achieving therapeutic CA levels in human tumors likely requires addressing the significant bioavailability challenge through formulation development.

Critical evaluation highlights several issues. The vast majority of evidence stems from *in vitro* cell lines, with limited *in vivo* data primarily from xenograft models (Zhang B. Y. et al., 2021; Chang et al., 2015; Ma et al., 2018; Cui et al., 2023; Horlad et al., 2013; Luo et al., 2024) that often poorly predict human clinical efficacy. Reported IC50 values vary widely, and physiologically relevant concentrations achievable *in vivo* are often unclear. Direct target engagement (e.g., binding affinities) is frequently suggested by docking but rarely confirmed biochemically (Zhang B. Y. et al., 2021; Chang et al., 2015; Jedram et al., 2023; Wu et al., 2023). Mechanisms appear highly context-dependent (cell type, species). For instance, CA induces apoptosis in many lines but non-apoptotic death in others (Jasim et al., 2023; Woo et al., 2018). Its role in ROS generation seems contradictory - inducing ROS in some settings (Nho et al., 2013; Jin et al., 2021; Woo et al., 2018) while potentially reducing it to overcome chemoresistance in others (Jin et al., 2021). AMPK activation is cited in gastric cancer (Lee et al., 2010b) but other pathways dominate elsewhere. Effects on macrophage polarization vary (Fujiwara et al., 2011; Horlad et al., 2013; Zhao et al., 2022). The relevance of ferroptosis sensitization (Peng et al., 2022; Jin et al., 2024) needs broader confirmation. The *in vivo* impact of CA metabolites (Zhang et al., 2019) is largely unexplored. Key controversies and gaps remain: Is there a unifying anti-cancer mechanism, or does CA act opportunistically? How does CA selectively target cancer cells over normal cells (often claimed but poorly substantiated (Cui et al., 2023; Luo et al., 2024))? What are the *bona fide* direct molecular targets? How effectively can CA penetrate solid tumors *in vivo*? Does CA interfere with or synergize with standard therapies (limited data (Fujiwara et al., 2013; Horlad et al., 2013; Lee et al., 2015; Park et al., 2018))? Determining the dominant anti-cancer pathway (e.g., AMPK’s role (Park et al., 2018)) requires further work. Dose optimization (adjuvant vs. cytotoxic) and addressing the bioavailability barrier are crucial. Potential off-target effects on normal cells and long-term toxicity require investigation. Minor inconsistencies, such as varying reports on autophagy’s role (Park et al., 2018) or NF-κB activation dynamics, need clarification, potentially relating to temporal effects or context. Table 3 summarizes the anti-tumor mechanisms of CA.

**TABLE 3:** Anti-tumour mechanism of CA (*In vitro* and *vivo*).

Cancer	Cell	Dosages (μM)	Mechanisms	Bibliography
*In vitro*				
Hepatocellular carcinoma	Bel-7404, Bel-7402	40	Enhancement of YAP phosphorylation by LATS1 accelerates YAP degradation and promotes βTrCP-dependent YAP ubiquitination	Xu et al. (2017)
	HepG2, Huh7, Hep3B	40	Translocation of YAP from the nucleus of HCC cells in the presence of MDM2	Jia et al. (2020)
	Bel-7404, HepG2	10; 20; 30	Inhibited the proliferation of the HCC cell lines Bel-7404 and HepG2 through activation of PERK-HCL, promoted cell death, and reduced the P4HA2 protein level in a dose-dependent manner	Tang et al. (2023a)
	Huh7	2.5	Inhibited VEGFR2 kinase activity by directly interacting with the ATP-binding pocket, downregulated the VEGFR2/Src/FAK/cdc42 axis, decreased the formation and *in vitro* migratory activity of F-actin	Chang et al. (2015)
Primary liver cancer	Bel-7402 and Bel-7404	10	Inhibited GSH through HERPUD1 *in vitro*, lowering the cellular GSH level and sensitizing hepatocellular carcinoma cells to iron apoptosis	Peng et al. (2022)
	Bel-7402; HepG2	20	Activated the PERK-eIF2a-ATF4 pathway, triggered ER stress-mediated apoptosis of HCC cells	Tang et al. (2023b)
Diabetes-associated liver cancer	Bel-7402; Bel-7404	40	Inactivated the CDK19/YAP/O-GlcNAcylation pathway through the regulation of the protein level, which reduced the cell proliferation ability of HCC cells in the high glucose environment and inhibited the tumor growth	Zhang et al. (2021b)
Cholangiocarcinoma	KKU-213A, KKU-213B	15,20,25	Inhibited the proliferation of KKU-213A and KKU-213B CCA cells and triggered apoptosis by altering the mitochondrial membrane potential (ΔΨm), increasing the Bax/Bcl-2 expression ratio, cytochrome c release, and caspase-3 activity	Jedram et al. (2023)
Gastric cancer	SNU-601 human gastric cancer cells	16.9 ± 2.9	Inhibited SNU-601 human gastric cancer cells; activated AMPK, triggered Caspase-3 and ADP-ribose polymerase; inhibited mTOR	Lee et al. (2010b)
	SNU-620	40.6	Enhanced the anticancer activity of 5-FU by inhibiting mTOR in SNU-620 human gastric cancer cells	Lee et al. (2015)
	NCI-N87 cells SNU-601 SNU-484	26.8 ± 7.9 43.7 ± 4.2 39.4 ± 7.7	Inhibited HER2 expression; inhibited the proliferation of HER2-positive gastric cancer cells and induced G (0)/G (1) arrest by inducing the downregulation of p27 (kip1) and cell cycle protein D (1)	Lee et al. (2010a)
	human gastric cancer cell line BGC823 cells	55.93 ± 7.34 μg/mL at 48 h 24.93 ± 3.52 μg/mL at 72 h	Inhibited the nuclear entry level of p65 and suppressed the expression level of NF-κB (p65) in a dose-dependent manner	Cheng et al. (2017)
	5-FU-resistant SNU-620/5-FUR cells	25	Activation of AMPK phosphorylation and reduction of TS expression and mTOR/4-EBP1 phosphorylation in 5-FU-resistant SNU-620/5-FUR cells; activation of the AMPK pathway sensitizes 5-FU-resistant gastric cancer cells and inhibits viability of 5-FU-resistant gastric cancer cells	Park et al. (2018)
Colorectal cancer	β-catenin and β-actin	40	Reduction of intracellular beta-catenin levels and inhibition of colon cancer growth in APC-mutant spontaneous mice; promotion of N-terminal phosphorylation and subsequent proteasomal degradation of b-conjugated proteins	Kim et al. (2014)
	HCT116 human colon cancer cells	24	CA-induced apoptosis was accompanied by activation of caspase-8, -9, and -3; which upregulated the pro levels of apoptosis proteins and downregulates the levels of anti-apoptosis proteins	Sung et al. (2014)
	HCT116 and SW480 cells	20	Modulation of Rala/RalBP1/CDK1 and PI3K/Akt/PKA pathways, directly targeting of HER2 and HER3 heterodimers, inhibition of mitochondrial fission; reduction of HER2 and HER3 phosphorylation in tumor tissues	Zhang et al. (2021a)
	human umbilical vein endothelial cells (HUVECs) and human dermal lymphatic microvascular endothelial cells (HDLMECs)	0, 5	Decreased phosphorylation of FAK and ERK1/2 and inhibition of proliferation and migration in human umbilical vein endothelial cells stimulated by angiopoietin-1	Yoo et al. (2015)
	HCT116 HT29 SW480 CCD-18Co	12.40 ± 1.05 μg/mL 16.35 ± 1.15 μg/mL 13.89 ± 1.06 μg/mL 27.58 ± 1.35 μg/mL	Inhibited NF-κB and ERK1/2 signaling and had a strong binding affinity for COX2, thereby reducing COX2 expression	Han et al. (2024)
Pancreatic cancer	HAPC and SW1990 cells	35	Inhibited the viability of PC cells and promoted the release of LDH; activated OS damage-induced apoptosis and senescence in HAPC and SW1990 cells	Luo et al. (2024)
	Capan-1 Bxpc-3 cells	Capan-1:15 μg/mL (proliferation); 15 μg/mL (migration) Bxpc-3:30 μg/mL (proliferation); 15 μg/mL (migration)	Induced spermine/spermine N1-acetyltransferase 1 - dependent iron metamorphosis and inhibited the migration and proliferation of Capan-1 cells	Jin et al. (2024)
Lung cancer	human lung adenocarcinoma A549 cell line	65	Inhibited the migration of A549 cells; inhibited the activity of VEGFR2 kinase and disrupted the structure of microtubule proteins	Li et al. (2019)
Human lung epithelial cancer	A549 human lung epithelial cancer cells	12 μg/mL at 48 h	Inhibited the growth of A549 human lung epithelial cancer cells under hypoxic conditions	Bahadori et al. (2019)
Non-small-cell lung cancer	A549 cells (invation; migration) PC9 cells (invation; migration)	A549: 58; 32 PC9: 26; 16	Reduced the level of the TPX 2 target protein, which inhibited the PI3K/AKT signaling pathway and induced apoptosis; reduced OS in mitochondria and liposomes by inducing NSCLC cell invasion and proliferation as well as chemoresistance	Jin et al. (2021)
Human lung adenocarcinoma	A549 cells	27.86	Causes dendritic enzyme-dependent apoptosis by altering anti-apoptotic proteins	Nho et al. (2013)
Bladder cancer	SW780	0, 6, 7, 8 μg/mL	Low concentration (<7.0 μg/mL) inhibited DNA synthesis mainly through downregulation of TOP2A and LIG1, and attenuated mitosis by down-regulating CCNA2, CCNB1, CDC20, and RRM2 High concentrations (≥7.0 μg/mL) of CA induced cell death by activating mitochondrial autophagy through the upregulation of NBR1, TAXBP1, SQSTM1/P62, and UBB	Cui et al. (2023)
Renal cancer	Caki cells: ACHN/A498/MDA-MB231/SK-Hep1/Huh7	2.5, 5, 10	Decreased cell viability and increased cytotoxicity in RCC cell Caki; CA-induced ROS levels were markedly elevated	Woo et al. (2018)
Renal cell carcinoma	786-O, ACHN, Caki-1, and HK2 cells Caki-1 cells	2, 4, 8, 12, 16 0, 2, 4, 8	Reduced MMP2 expression; MMP2 expression decreased in a dose-dependent manner with increasing CA concentration, and CA also stimulated the phosphorylation of ERK1/2 in 786-O and Caki-1 cells	Wu et al. (2023)
Prostate cancer	PC-3; DU145; 22RV1; WPMY-1	PC-3: 14.34 (24 h); 9.769 (48 h) DU-145: 17.21 (24 h); 8.738 (48 h) 22RV1: 18.41 (24 h); 11.64 (48 h) WPMY-1: 68.36 (24 h); 36.22 (48 h)	Inhibited the growth of PCa and DU145 cell lines, significantly activated two pro-apoptotic signaling pathways (MAPK and AKT signaling pathways) associated with ENR stress and induced apoptosis	Ma et al. (2018)
	TRAMP-C1 cells	2, 4, 8	Inhibited the dose-dependent growth of prostate cancer TRAMP-C1 cells *in vitro*; CA induced the mRNA and protein expression of Nrf2, HO-1, and NQO1; and increased the expression of H3K27ac acetylation and decreased H3K27me3	Yang et al. (2018)
Advanced epithelial ovarian cancer	SKOV3, RMG-1, and ES-2	10, 20, 30, 40, 50	Reversed the chemoresistance of epithelial ovarian cancer cells, inhibited the interaction between tumor cells and tumor-associated macrophages, inhibited macrophage-induced activation of epithelial ovarian cancer cells	Katsura et al. (2022)
Breast cancer	MDA-MB-231 cell lines MCF7 cell lines	20.12 28.50	Induced apoptosis in MADA-MB-231 cells by decreasing the phosphorylation of JAK-2-and-STAT 3, and induced the death of these cells by stimulating the apoptotic pathway and inhibiting JAK/STAT -3 signaling triggered the death of these cells	Fujiwara et al. (2013)
	sPLA2IIa	9.44 ± 0.59	The inhibitor of sPLA2IIa, potentially neutralized its indirect hemolytic activity and paw edema in mice	Pundalik et al. (2022)
Skin cancer	Cox-2, Il17a, Il22, Cxcl1, Cxcl2, and Vegfa	2	Inhibited cell proliferation, skin inflammation, and expression of inflammatory genes *in situ* skin cancers induced by the tumor-promoting agent TPA, and reduced EGFR-IGF, STAT3-Twist1, Cox-2, and AMPKa	Cho et al. (2015)
	JB6P+ cells	2.5–15	Reversed TPA-induced DNACpG methylation of CDK1 and RASSF2 in mouse epidermal epithelial JB6 P+ cells	Hudlikar et al. (2020)
Retinoblastoma	human retinoblastoma Y-79 cells	4.15 (24 h) 3.37 (48 h)	Had dose-dependent cytotoxicity, cell cycle arrest, and apoptosis-inducing effects on human retinoblastoma Y-79 cells, and MELK-FoxM1 signaling was involved in the CA cytotoxicity on Y-79 cells	Wang et al. (2018)
Human oral squamous cell carcinoma	HSC3 and SAS cells	2.5, 5, 10, 15, 20	Inhibits OSCC by suppressing cell progression on the ERK1/2-MMP1 axis	Chen et al. (2021)
Osteosarcoma	osteosarcoma MG-63 cells	33.7 (24 h) 23.6 (48 h)	Disrupted the mitochondrial membrane potential, triggering the release of cytochrome c from mitochondria into the cytoplasm, as well as triggering the activation of caspases 8, 9, and 3	Cai et al. (2011), Jia et al. (2016)
	Saos2, HSOS-1, LM85	10, 20	Inhibition of signal transduction and activation of Stat3 Reduced the expression of cyclic oxidase-2 and CCL2 mRNA	Horlad et al. (2013)
Ascites carcinoma	CD163; IL-10; U373 and T98G	30	Inhibited the proliferation of glioblastoma cells U373 and T98G; inhibited the activation of STAT3 and NF-κB	Fujiwara et al. (2011)
Glioblastoma	CTX-TNA2 and GBM cell lines (GBM8401, M059K, U251-MG and U87-MG)	10, 15, 20, 25, 30	Promoted ubiquitin-mediated proteasomal degradation by upregulation of E3 ligases and the CHIP to promote AXL degradation and inhibit GBM cell migration and invasion; downregulated the expression level of GAS6 and inhibited the phosphorylation of JAK2, MEK, and ERK	Sun et al. (2021)

Cancer	Models	Dosage (mg/kg)	Controls (positive and negative)	Mechanisms	Bibliography
*In vivo*					
Hepatocellular carcinoma	Xenograft mice (HEPG2-induced)	20, i.p., qod	Neg: DMSO	Translocation of YAP from the nucleus of HCC cells in the presence of MDM2	Zhu et al. (2022)
	NOD/SCID mice	10, i.p	Neg:DMSO	Inhibited VEGFR2 kinase activity by directly interacting with the ATP-binding pocket, downregulated the VEGFR2/Src/FAK/cdc42 axis, decreased the formation and *in vitro* migratory activity of F-actin	Ansari et al. (2021)
Primary liver cancer	SD rats with associating liver partition and portal vein ligation	20, i.p, qod	Neg: water	Induced downregulation of PD-1 and TGF-βexpression and inhibited M2 macrophage poles	El-Hawary et al. (2022)
	Bel-7402-Xenograft mice	10, i.h	Pos: Imidazole ketone erastin	Inhibited GSH through HERPUD1 *in vitro*, lowering the cellular GSH level and sensitizing hepatocellular carcinoma cells to iron apoptosis	Peyeino et al. (2022)
Diabetes-associated liver cancer	Bel-7404-Xenograft mice	10 *via* intratumoral injection	Neg: methanol	Inactivated the CDK19/YAP/O-GlcNAcylation pathway through the regulation of the protein level, which reduced the cell proliferation ability of HCC cells in the high glucose environment and inhibited the tumor growth	Albuquerque et al. (2020)
Colorectal cancer	HCT116 xenograft model (AOM-DSS model)	10 or 30 g, i.g	Neg: AOM-DSS without drugs	Modulation of Rala/RalBP1/CDK1 and PI3K/Akt/PKA pathways, directly targeting of HER2 and HER3 heterodimers, inhibition of mitochondrial fission; reduction of HER2 and HER3 phosphorylation in tumor tissues	Wang et al. (2014)
	CT-26 allograft colon carcinoma animal model	5, 25 *via* intratumoral injection	Neg: PBS with 0.2% DMSO	Decreased phosphorylation of FAK and ERK1/2 and inhibition of proliferation and migration in human umbilical vein endothelial cells stimulated by angiopoietin-1	Chen et al. (2014)
Pancreatic cancer	HPAC-induced xenograft tumor model	15 mg/kg, 3 times/week) for 2 weeks, i.p	Neg: DMSO	Inhibited the viability of PC cells and promoted the release of LDH; activated OS damage-induced apoptosis and senescence in HAPC and SW1990 cells	Yao et al. (2013)
	Panc-1-induced xenograft tumor model	2.5, i.g	​	Induced spermine/spermine N1-acetyltransferase 1 - dependent iron metamorphosis and inhibited the migration and proliferation of Capan-1 cells	Li et al. (2012)
Lung cancer	A549-induced xenograft tumor model	2, 4, 8	Neg: 10% DMSO	Inhibited the migration of A549 cells; inhibited the activity of VEGFR2 kinase and disrupted the structure of microtubule proteins	Liu et al. (2009)
Non-small-cell lung cancer	NSCLC-induced xenograft tumor model	1.5, 2.5, 3	Neg: PBS	Reduced the level of the TPX 2 target protein, which inhibited the PI3K/AKT signaling pathway and induced apoptosis; reduced OS in mitochondria and liposomes by inducing NSCLC cell invasion and proliferation as well as chemoresistance	Aladedunye et al. (2008)
Bladder cancer	SW780-induced xenograft tumor model	8, i.p. qod	Neg: DMSO	Low concentration (<7.0 μg/mL) inhibited DNA synthesis mainly through downregulation of TOP2A and LIG1, and attenuated mitosis by down-regulating CCNA2, CCNB1, CDC20, and RRM2 High concentrations (≥7.0 μg/mL) of CA induced cell death by activating mitochondrial autophagy through the upregulation of NBR1, TAXBP1, SQSTM1/P62, and UBB	Liu et al. (2020)
Prostate cancer	PC-3-induced xenograft tumor model	10, 20, intraperitoneal injection,qod	Neg: 10% DMSO+ 90% Saline	Inhibited the growth of PCa and DU145 cell line, significantly activated two pro-apoptotic signaling pathways (MAPK and AKT signaling pathways) associated with ENR stress and induced apoptosis	Rao et al. (2012)
Breast cancer	EAC-induced xenograft tumor model	10, p.o	Pos: 5-FU Neg: PBS	The inhibitor of sPLA2IIa, potentially neutralized its indirect hemolytic activity and paw edema in mice	Takagi et al. (2010)
Osteosarcoma	LM-85-induced xenograft tumor model	17.5	Neg: 0.5% methylcellulose	Inhibition of signal transduction and activation of Stat3 Reduced the expression of cyclic oxidase-2 and CCL2 mRNA	Fujiwara et al. (2011)

### 3.3 Anti-inflammatory, antioxidant, and cardiovascular effects

CA exhibits significant anti-inflammatory actions, potentially mediated through targeting key signaling pathways like IKKβ/NF-κB (Gautam et al., 2015; Balakrishnan and Al-Assaf, 2016; Wang et al., 2020; Zhang et al., 2020; Chen et al., 2012) and STAT3 (Fujiwara et al., 2011; Horlad et al., 2013; Kawade et al., 2023; Yamamura et al., 2024; Andersen et al., 2019). It has also been shown to inhibit NLR family pyrin domain containing 3 (NLRP3) inflammasome activation (Li Y. et al., 2016). Its antioxidant effects are linked to the activation of the Nrf2/heme oxygenase-1 (HO-1) pathway (Yang et al., 2018; Sahu et al., 2014; Peng et al., 2019) and the inhibition of NADPH oxidase 2 (NOX2) (Li Y. et al., 2016). In the cardiovascular system, CA demonstrates multiple benefits including vasodilation (potentially via nitric oxide (NO)/cyclic guanosine monophosphate (cGMP) and hydrogen sulfide (H_2_S)/ATP-sensitive potassium channel (KATP) pathways) (Luna-et al., 2018; Torres-Ortiz et al., 2019), amelioration of atherosclerosis (linked to monocyte chemoattractant protein-1 (MCP-1) inhibition) (Chen et al., 2012; Li et al., 2022), improvement in models of pulmonary hypertension (associated with STAT3 inhibition) (Kawade et al., 2023; Yamamura et al., 2024), protection against myocardial injury and hypertrophy (via mechanisms involving Nrf2 activation (Sahu et al., 2014), AMPK-autophagy regulation (Wang et al., 2019), peroxisome proliferator-activated receptor gamma (PPAR-γ) activation (Alkholifi et al., 2023), and inhibition of transforming growth factor beta (TGF-β)/NF-κB signaling (Wang et al., 2020)), and protection against cerebral ischemia-reperfusion injury (Zhang et al., 2022). In the context of joint inflammation, CA may inhibit osteoclastogenesis (potentially involving JNK/AMPK/Nrf2 signaling) (Peng et al., 2019), protect chondrocytes from degradation (possibly by activating autophagy via the PI3K/AKT/mTOR pathway) (Han et al., 2022), and inhibit interleukin-1 receptor-associated kinase 1 (IRAK-1) (Kim et al., 2016).

Critically evaluating this evidence, the inhibition of NF-κB and STAT3 appears relatively consistent across different inflammatory models. Nrf2 activation provides a plausible antioxidant mechanism. However, direct target engagement (e.g., with IKKβ, STAT3) often relies on downstream readouts or *in silico* data rather than direct biochemical confirmation (Gautam et al., 2015). Cardiovascular protection studies utilize diverse, often acute, injury models (e.g., isoproterenol, doxorubicin, monocrotaline, STZ induction) (Alkholifi et al., 2023; Kawade et al., 2023; Sahu et al., 2014; Che et al., 2021), and the relevance of findings from these models to chronic human cardiovascular conditions needs careful consideration. A key limitation is distinguishing CA’s direct effects from secondary consequences of metabolic improvement, particularly in conditions like diabetic cardiomyopathy (Alkholifi et al., 2023). The relative contributions of anti-inflammatory versus antioxidant versus direct signaling modulation (e.g., AMPK) to cardioprotection are not well dissected. Furthermore, *in vivo* validation for proposed vasodilation mechanisms is limited (Luna-et al., 2018). Major gaps include identifying the primary pathway driving CA’s benefits in specific cardiovascular or inflammatory conditions (NF-κB, STAT3, AMPK, Nrf2, PPAR-γ). How CA selectively modulates these pathways (e.g., inhibiting pro-inflammatory STAT3 in macrophages (Andersen et al., 2019) while potentially impacting it differently elsewhere) is unclear. Finally, whether the effects observed in acute injury models are sustainable in chronic disease settings remains an open question.

### 3.4 Current clinical evidence: an overview of human studies and safety

Despite the extensive preclinical data demonstrating the multi-target potential of CA in regulating glucose metabolism, high-quality human evidence supporting its clinical translation is extremely scarce, constituting the most significant “translational gap” in current research. The existing directly relevant evidence primarily comprises three key studies, which exhibit substantial differences in type, depth, and clinical relevance.

A trial published in 2006 (94) provides preliminary yet crucial human validation for CA’s effect. This study employed a randomized, double-blind, placebo-controlled, crossover design involving 31 subjects (including 19 diabetic patients). Subjects received a single oral dose of 10 mg CA 5 min before a 75g oral glucose tolerance test. The results showed that compared to the placebo, the CA group began to exhibit lower blood glucose levels starting at 60 min post-glucose administration, reaching a statistically significant difference at 90 min, with this effect persisting until 120 min. However, this study had a limited sample size, involved only a single dose, and failed to assess long-term efficacy or systematically collect safety data. Another cell-based mechanistic study published in 2008 (93) found that in L6 rat skeletal muscle myotubes, a 250 nM concentration of CA could reduce the half-maximal effective concentration (EC_50_) for insulin-stimulated glucose uptake by approximately 5.6-fold and enhance insulin receptor phosphorylation. This provides an *in vitro* mechanistic explanation for CA’s “plant insulin”-like action, but this finding has not yet been directly confirmed in humans. Furthermore, a 2011 systematic review (Stohs et al., 2012) concluded that the use of standardized Banaba (*Lagerstroemia speciosa* L.) leaf extract containing CA could induce a dose-dependent decrease in blood glucose in patients with type II diabetes and was generally “well-tolerated”. However, the original studies on which this conclusion relies were insufficient in terms of design details, sample size, and reporting of specific safety data.

Based on the above evidence, the current clinical translation pathway for CA faces the following fundamental limitations: (1) Key human evidence is isolated and outdated: The only rigorously designed human trial was published nearly 2 decades ago. Its conclusion (that CA can lower postprandial blood glucose) has not yet been validated and expanded upon by subsequent larger-scale, longer-duration clinical trials, creating the most prominent break in the evidence chain. (2) Severe disconnect between mechanism and clinical effect: There is a lack of a bridge between the abundant preclinical mechanistic discoveries (e.g., enhanced insulin sensitivity) and the extremely limited human pharmacodynamic data. Key questions such as the dominant pathways of action for CA in humans and effective blood concentration levels remain unknown. (3) Complete absence of long-term safety and efficacy data: None of the studies have addressed whether CA can sustainably reduce HbA1c, a core efficacy indicator in diabetes management. There is also a complete lack of safety assessment for long-term use. (4) Deficiency in pharmacokinetic and formulation research: Key pharmacokinetic parameters of CA in humans, such as bioavailability, half-life, and active metabolites, have not been systematically elucidated. Furthermore, the raw materials and formulations used in existing studies are not uniform, rendering the determination of a clinically effective dose.

In summary, the current evidence can only suggest that CA has the potential to regulate glucose metabolism but is far from sufficient to establish its clinical value. Between the vast amount of *in vitro* mechanistic data and the sporadic, outdated, and incomplete human data lies a “translational canyon” that urgently needs to be crossed. The primary direction for future research must be the conduct of well-designed early-phase clinical trials to obtain reliable clinical pharmacodynamic, pharmacokinetic, and safety data.

### 3.5 Other biological effects and formulation strategies

Preliminary studies suggest additional biological activities for CA. It exhibits anti-microbial activity and may enhance the efficacy of antibiotics like cefotaxime against *Staphylococcus aureus* (Sinelius et al., 2023; Zhou et al., 2020; Abdelmalek et al., 2021). Anti-viral potential has also been proposed based on *in silico* studies (Vardhan and Sahoo, 2020). Anti-aging and skin benefits, such as stimulation of collagen and hyaluronic acid production, have been suggested (Tan et al., 2017). Furthermore, CA can inhibit drug metabolism by interfering with enzymes like cytochrome P450 3A (CYP3A) (Kim et al., 2010). Recognizing the challenge posed by CA’s poor water solubility (Qian et al., 2021) and consequent low bioavailability, significant effort has been directed towards developing advanced formulation strategies to enhance its delivery. Various approaches have been explored, including the synthesis of water-soluble glycoside derivatives (Xu et al., 2016), encapsulation in liposomes (including targeted versions, e.g., anti-Cluster of Differentiation 163 (CD163) for macrophages (Andersen et al., 2019), and drug-loaded systems (Widjaya et al., 2022; Li et al., 2020)), complexation with cyclodextrins (Bao et al., 2022), development of self-assembling systems (Bag et al., 2019), formulation into lipid nanoparticles (Guo et al., 2023), and creation of SMEDDS (96). Among these, corosolic acid di-lactoside (IC_50_ = 428 μM) exhibits superior α-glucosidase inhibitory activity compared to acarbose (IC_50_ = 478 μM) and demonstrates better water solubility (Xu et al., 2016). Unfortunately, most of the aforementioned articles fail to specify precise improvement effects, relying instead on vague descriptions such as “significantly enhanced” or “notably improved”. Furthermore, these studies were conducted solely at the cellular level without further *in vivo* experimental research.

Evaluation of these findings indicates that the anti-microbial and anti-aging data are largely preliminary and derived from *in vitro* studies. Formulation studies have successfully demonstrated improved solubility or cellular uptake (Xu et al., 2016; Li et al., 2020; Guo et al., 2023), and in some cases, enhanced *in vivo* efficacy in animal models compared to free CA (Agarwal et al., 2018; Widjaya et al., 2022). However, enhanced delivery does not automatically guarantee clinical efficacy if the intrinsic activity or safety profile in humans is insufficient. A significant limitation is the general lack of robust comparative PK/PD data for these novel formulations versus free CA, and between different formulation types. The long-term stability and potential toxicity of nanoparticle and liposomal formulations require thorough assessment. Key research gaps include understanding whether these formulations significantly alter the *in vivo* mechanism of action or metabolite profile of CA. The feasibility and clinical benefit of targeted delivery strategies (e.g., anti-CD163 liposomes (Andersen et al., 2019)) beyond preclinical models remain to be established. Additionally, whether advanced formulation strategies can help overcome potential off-target effects associated with systemic exposure to CA is an important question for future investigation.

## 4 Discussion

CA presents a compelling case as a natural compound possessing remarkably broad preclinical bioactivity. Its documented effects encompass metabolic regulation, inhibition of cancer cell proliferation and survival, control of inflammation, and cardiovascular protection. These diverse activities appear to be mediated through the modulation of central cellular signaling hubs, including AMPK, NF-κB, STAT3, YAP, and various receptor tyrosine kinases (RTKs). This discussion aims to synthesize the proposed mechanisms, address key controversies and knowledge gaps, outline current research limitations, and propose a roadmap for future investigation towards potential clinical translation.

### 4.1 Mechanistic integration and complexity

The pleiotropic effects observed for CA likely stem from its ability to influence interconnected signaling networks, bridging metabolic regulation and oncogenic signaling pathways. A unifying theme appears to be the activation of energy-sensing and stress-response pathways coupled with the simultaneous suppression of pro-growth and pro-inflammatory signals.

AMPK emerges as a potential central node in CA’s mechanism of action. Its activation has been reported in diverse contexts (Yang et al., 2016a; Liu et al., 2021; Wang et al., 2019; Lee et al., 2010b; Li Y. et al., 2016; Peng et al., 2019), reflecting its origin as a compound noted for glycemic control, sometimes termed a “phyto-insulin.” AMPK activation can trigger a cascade of downstream effects potentially explaining CA’s broad bioactivity: it could simultaneously improve insulin sensitivity, suppress hepatic lipogenesis (partially via SREBPs) (Zhang et al., 2020), inhibit the mTOR pathway contributing to anti-cancer effects (Lee et al., 2010b), promote autophagy relevant for cardioprotection and chondroprotection (Wang et al., 2019; Han et al., 2022; Che et al., 2021), enhance fatty acid oxidation, promote autophagic clearance of lipid droplets, and potentially dampen inflammation, although its interactions with NF-κB and STAT3 are complex and context-dependent. This capacity connects nutrient status to multiple cellular processes, providing a rationale for CA’s dual benefits in metabolic disorders and cancer suppression, as improved lipid/glucose handling, reduced lipotoxic stress, and lower inflammation are favorable in both settings like type 2 diabetes (T2D) and tumor development.

Furthermore, CA intercedes in key pathways governing the inflammation-cancer-metabolism axis. Inhibition of pro-inflammatory transcription factors like NF-κB and STAT3 (Gautam et al., 2015; Balakrishnan and Al-Assaf, 2016; Wang et al., 2020; Fujiwara et al., 2011; Chen et al., 2012) may contribute not only to direct anti-inflammatory effects (e.g., reducing pro-inflammatory cytokines like TNFα and IL-6 in adipose or liver tissue) but also significantly impact cancer progression, as chronic inflammation is known to fuel many cancers, and metabolic dysfunction (metaflammation). By repressing NF-κB/STAT3 activity, CA might improve insulin sensitivity while concurrently depriving tumor cells of crucial survival signals and potentially promoting immune-mediated tumor clearance. Similarly, CA’s ability to modulate PPARs and other metabolic regulators may underlie its insulin-sensitizing effects in diabetes as well as its anti-proliferative impact on cancer cells, given that metabolic reprogramming is a hallmark of cancer. Many of these pathways converge, suggesting CA targets the common pathological link of metabolic inflammation connecting metabolic syndrome with cancer risk.

Beyond metabolic and inflammatory pathways, CA interferes with growth factor signaling and specific oncogenic pathways. This includes interference with RTKs such as VEGFR2, HER2/3, and EGFR (Zhang B. Y. et al., 2021; Chang et al., 2015; Lee et al., 2010a; Li et al., 2019; Cho et al., 2015), potentially impacting tumor angiogenesis and proliferation. Recent studies also highlight interference with oncogenic modules like the Hippo-YAP pathway (Xu et al., 2017; Jia et al., 2020; Zhang C. et al., 2021). For instance, in HCC models, CA was reported to affect β-TrCP-mediated ubiquitination of YAP, paradoxically resulting in elevated YAP levels alongside reduced β-TrCP expression, while concurrently downregulating VEGFR2/Src/FAK signaling, linking CA to the control of tumor cell motility and angiogenesis. In other cancer contexts, CA reportedly destabilizes oncogenic receptors, such as promoting AXL degradation in GBM (inhibiting the GAS6/AXL axis) and disrupting HER2/HER3 dimerization in CRC. CA can induce apoptosis via intrinsic mitochondrial pathways (triggering classical caspase-dependent apoptosis in many tumor lines) and halt cell cycle progression, yet it also appears capable of inducing non-apoptotic cell death through mechanisms like lipid peroxidation in certain contexts (Woo et al., 2018; Al-Medhtiy et al., 2024; Thakur and Devaraj, 2020). This highlights multiple avenues for eliminating cancer cells.

Finally, the modulation of redox balance, including effects on ROS (Nho et al., 2013; Jin et al., 2021; Woo et al., 2018) and activation of the Nrf2 pathway (Yang et al., 2018; Sahu et al., 2014; Peng et al., 2019), likely contributes to cytoprotection in normal tissues (e.g., cardiovascular, neuroprotection) but may also sensitize cancer cells to death, potentially involving mechanisms like ferroptosis or specific types of apoptosis.

In aggregate, these mechanistic insights portray CA as an agent that integrates metabolic and oncogenic pathway inhibition. It seemingly aligns metabolic homeostasis (through AMPK activation, lipid/autophagy regulation) with direct anti-tumor actions (via NF-κB/STAT3 inhibition, pro-apoptotic signaling, interference in oncogenic kinases and redox modulation). This integrative mechanism is especially relevant for diseases like T2D and certain cancers (e.g., HCC) where chronic metabolic stress, inflammation, and malignant transformation are interlinked. CA’s capacity to simultaneously alleviate metabolic dysfunction and restrain malignant cell signaling exemplifies a multi-faceted approach, potentially addressing the metabolism-tumor continuum at several levels.

### 4.2 Key controversies and knowledge gaps

Despite the extensive preclinical data and proposed connections, critical questions, inconsistencies, and knowledge gaps persist, fueling ongoing debate and highlighting areas requiring further investigation.

One major area of uncertainty concerns the direct versus indirect targets of CA. While numerous downstream effects are documented, robust evidence identifying the primary proteins CA directly binds to initiate these cascades is often lacking. Much current evidence relies on *in silico* docking simulations or inferring targets from downstream pathway readouts, which can be misleading. Target deconvolution using unbiased biochemical and proteomic methods is crucial to definitively establish the initiating molecular interactions. Relatedly, there is debate regarding whether CA possesses a specific “druggable” primary target or if its efficacy stems from broad polypharmacology. Unlike targeted therapies, CA influences multiple pathways (AMPK, NF-κB, STAT3, YAP, EGFR/HER2, etc.), making it challenging to pinpoint a singular initiating mechanism. Some argue this multi-target nature is advantageous for complex diseases, while others express concern that the lack of a defined primary target hinders optimization and prediction of off-target effects. The absence of direct binding data means mechanisms are often inferred, leading to a patchwork of potentially inconsistent claims across studies (e.g., varied explanations for glucose lowering or apoptosis induction). The similarity of CA to other pentacyclic triterpenoids also raises questions about its unique mechanistic signature versus representing a broader class effect.

Context-dependency and signaling paradoxes represent another significant challenge. Why CA activates AMPK prominently in some cellular or disease contexts while other pathways dominate elsewhere, or why it induces different forms of cell death (e.g., apoptosis vs. ferroptosis-like death), is not fully understood. Understanding the influence of the specific cellular milieu and disease state is paramount. A specific example is the paradoxical finding regarding CA’s effect on the Hippo-YAP pathway in HCC cells, where it reportedly increased YAP protein levels (typically pro-tumorigenic) via β-TrCP suppression. The interpretation remains ambiguous - whether this stabilizes a growth promoter or triggers a context-specific cell death feedback loop, possibly requiring co-treatments like Actinomycin D as suggested by some authors. This exemplifies how outcomes can vary, fueling debate on the significance and intentionality of certain pathway modulations. Literature variability in assigning primacy to different signaling nodes (e.g., NF-κB vs. AMPK vs. MAPK) further fragments understanding and requires careful, context-specific interpretation.

The redox duality of CA is another point of controversy. Findings appear contradictory: in metabolic disease models, CA is often associated with antioxidant effects, reducing oxidative stress and enhancing defenses (e.g., upregulating SOD, glutathione). However, in certain cancer cells, CA seemingly acts as a pro-oxidant, inducing lethal levels of oxidative stress, such as the intense lipid peroxidation observed in HCC cells leading to non-apoptotic death resembling ferroptosis. This raises the fundamental question of whether CA is primarily an antioxidant whose effects become pro-oxidant in susceptible tumor cells, or if it possesses truly context-dependent biphasic redox behavior. Clarity on this is needed for accurate mechanistic classification.

Furthermore, the relative contribution of direct cytotoxicity versus indirect immunomodulation to CA’s anti-tumor effects remains ambiguous. While *in vitro* studies clearly show direct pro-apoptotic and anti-proliferative actions on isolated cancer cells, emerging *in vivo* evidence in immunocompetent models suggests significant remodeling of the tumor microenvironment. For example, reports indicate CA treatment can increase intratumoral CD4^+^ and CD8^+^ T-lymphocytes, abrogate MDSCs, and skew macrophages away from a tumor-promoting M2 phenotype, potentially linked to STAT3 inhibition in immune cells. The controversy lies in quantifying the relative importance of these direct versus immune-mediated mechanisms. The reliance on immunodeficient xenograft models in many studies may have underestimated the immunomodulatory component. Definitive experiments comparing outcomes in immune-competent versus deficient settings are needed to resolve whether CA should be developed primarily as a cytotoxic agent or potentially as an immunotherapeutic adjuvant.

Crucial gaps also exist regarding PK, PD, and the role of metabolites. What are the achievable plasma and tissue concentrations of CA and its metabolites (Zhang et al., 2019) in humans following administration? Are the metabolites more or less active, and do they significantly contribute to the observed effects *in vivo*? This remains a major blind spot, limiting translation.

Finally, the translational relevance of current models and the therapeutic window are significant unknowns. How predictive are the predominantly used *in vitro* assays and conventional animal models for human efficacy and safety? More sophisticated preclinical models are needed. Furthermore, the margin between potentially efficacious doses and toxicity, especially with chronic administration relevant for metabolic diseases or long-term cancer control, is largely undefined.

### 4.3 Limitations of current research landscape

The current body of research on CA, while extensive, suffers from several limitations that hinder definitive conclusions and clinical translation: (1) A predominance of descriptive *in vitro* studies, often in standard cell lines, which may not fully capture the complexity of human physiology or disease microenvironments. (2) A relative lack of rigorous mechanistic validation, particularly concerning the identification of direct molecular targets and the use of genetic or precise pharmacological tools to confirm pathway dependencies. (3) Limited utilization of advanced or highly relevant *in vivo* models, such as genetically engineered mouse models (GEMMs), patient-derived xenografts (PDXs), humanized mouse models, or models of chronic disease progression. (4) A scarcity of robust PK, PD, and metabolite profiling data, especially comparative data across species and relevant human data. (5) An absence of well-designed, adequately powered human clinical trials investigating efficacy for specific indications. (6) Potential publication bias favouring positive findings, which might skew the overall perception of CA’s efficacy and consistency. (7) A lack of standardized CA material (extraction, purification, quantification) and quality control across studies, potentially contributing to variability in reported results.

### 4.4 Future research roadmap: towards clinical translation

To bridge the significant translational gap and rigorously evaluate the therapeutic potential of CA, a multi-pronged research strategy is imperative, moving beyond descriptive studies towards robust mechanistic understanding and clinically relevant validation.

Fundamental Mechanistic Studies: Target Identification: Employ unbiased, state-of-the-art methods such as chemical proteomics (e.g., activity-based protein profiling), affinity chromatography coupled with mass spectrometry, or thermal proteome profiling to identify direct binding partners of CA in relevant cellular contexts. Key interactions must then be validated biochemically (e.g., using Surface Plasmon Resonance (SPR) or Isothermal Titration Calorimetry (ITC)) and functionally within cells.

Pathway dissection: Utilize precise genetic tools (e.g., CRISPR/Cas9 knockouts/knock-ins of specific isoforms or pathway components) and highly selective pharmacological inhibitors/activators to confirm the necessity and sufficiency of proposed signaling pathways (e.g., specific AMPK isoforms, NF-κB subunits, YAP/TAZ, key RTKs) in mediating the distinct biological effects of CA in relevant cell types (e.g., hepatocytes, adipocytes, specific cancer cells, immune cells).

Metabolite profiling & activity: Conduct comprehensive characterization of CA metabolites formed *in vivo* in relevant preclinical species (rodents, larger animals) and eventually in humans. Isolate or synthesize major metabolites and systematically test their individual bioactivities, PK profiles, and potential contribution to the overall therapeutic effect or toxicity profile.

#### 4.4.1 Improved preclinical models

Advanced *in vitro* models: Move beyond 2D monocultures to utilize more physiologically relevant systems like 3D cultures (spheroids, organoids), patient-derived cells, and co-culture systems that incorporate components of the tissue microenvironment (e.g., cancer cells co-cultured with fibroblasts, immune cells, or endothelial cells).

Relevant *in vivo* models: Progress beyond simple subcutaneous xenografts or acute injury models. Employ GEMMs that better recapitulate the genetic complexity and progression of human diseases, PDX models for evaluating efficacy in patient-derived tumor tissues, humanized mouse models (e.g., containing human immune system components) to study immune interactions, and models reflecting chronic disease states (e.g., long-term diet-induced obesity/diabetes models).

Robust pharmacokinetics and safety: PK/PD Studies: Perform thorough absorption, distribution, metabolism, excretion (ADME) studies in multiple species, including non-rodents if possible, to determine bioavailability (especially comparing different potential formulations), tissue distribution, clearance mechanisms, and establish clear relationships between dose, exposure (plasma/tissue concentrations), and PD markers of effect (e.g., pathway modulation biomarkers).

Toxicology: Conduct comprehensive short-term and long-term toxicology studies adhering to regulatory guidelines. Assess potential organ toxicities, off-target effects, genotoxicity, and establish a safe dose range and therapeutic index.

Standardization and quality control: Develop, validate, and implement rigorous standards for the extraction, purification, characterization, and quantification of CA used in research. Ensuring the consistency and quality of the investigational material is paramount for reproducibility across laboratories and studies.

Well-designed (pilot) clinical trials: phase 0/i trials: Once sufficient preclinical evidence (strong mechanistic rationale for a specific indication, robust efficacy in relevant models, adequate safety/toxicology data, defined PK/PD relationship) exists, initiate early-phase human trials (Phase 0 or I). These should primarily focus on safety, tolerability, and human PK. Incorporating PD biomarker assessments (e.g., measuring target engagement like p-AMPK or downstream effects like inflammatory markers in accessible tissues like blood or biopsies) can provide crucial proof-of-concept data.

Targeted indications: Prioritize clinical investigation for indications where the preclinical mechanistic evidence for CA’s action is strongest, most consistent, and where it might offer a distinct advantage or fulfill an unmet need compared to existing therapies. Examples could include metabolic syndrome with a significant inflammatory component, or specific cancer types shown to be reliant on pathways robustly modulated by CA in preclinical studies. Rationally designed combination therapies, based on mechanistic synergy identified preclinically, should also be considered for future trials.

By systematically addressing these mechanistic questions, controversies, and translational hurdles through rigorous, well-designed studies, the scientific community can better delineate the true therapeutic potential and limitations of CA.

## 5 Conclusion

As a multi-target therapeutic agent, CA holds considerable preclinical promise. However, it still exhibits the following limitations: 1) Existing studies are numerous but lack depth, with a general absence of rigorous mechanism validation and consistent data; 2) AMPK/NF-κB/STAT3/YAP serve as critical nodes, yet direct targets remain unidentified; 3) Limited data support regarding human PK/PD parameters, metabolite activity, long-term safety, and efficacy validation in advanced disease models.

**FIGURE 2:**
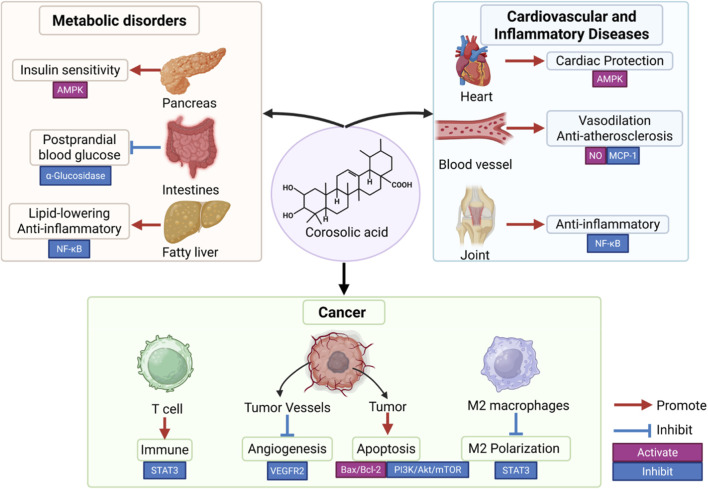
The primary mechanism of action of CA (Created in BioRender. https://BioRender.com/puyadcl).

Moving forward, the field must pivot from descriptive exploration to hypothesis-driven, mechanistically rigorous research using advanced models and standardized materials. Only through such a critical and structured approach, culminating in carefully designed clinical trials for well-justified indications, can the true therapeutic potential of CA be realized or refuted. The proposed roadmap provides a framework for navigating the complex path from preclinical hype to potential clinical reality. Additionally, exploring the potential of CA to treat multiple conditions via shared pathways warrants further exploration (Figure 2).

## Glossary

CA: Corosolic Acid
AMPK: AMP-activated protein kinase
NF-κB: Nuclear factor kappa B
YAP: Yes-associated protein
VEGFR2: Vascular endothelial growth factor receptor 2
HER2/3: Human epidermal growth factor receptor 2/3
PK: Pharmacokinetics
PD: Pharmacodynamics
PTP1B: Protein-tyrosine phosphatase 1B
MAPK: Mitogen-activated protein kinase
ERK: Extracellular signal-regulated kinase
JNK: C-Jun N-terminal kinase
AGEs: Advanced glycation endproducts
ACAT-2: Acyl-CoA:cholesterol acyltransferase-2
SREBPs: Sterol regulatory element-binding proteins
NAFLD: Non-alcoholic fatty liver disease
NASH: Non-alcoholic steatohepatitis
ALD: Alcoholic liver disease
SOCE: Store-operated calcium entry
Akt: Protein kinase B
GLUT4: Glucose transporter type 4
PI3K: Phosphoinositide 3-kinase
HFD: High-fat diet
IKKβ: Inhibitor of nuclear factor kappa-B kinase subunit beta
TNFα: Tumor necrosis factor alpha
IL-6: Interleukin-6
IRS-1: Insulin receptor substrate 1
ROS: Reactive oxygen species
SMEDDS: Self-microemulsifying drug delivery system
HbA1c: Glycated hemoglobin A1c
HCC: Hepatocellular carcinoma
CCA: Cholangiocarcinoma
CRC: Colorectal
RB: Retinoblastoma
GBM: Glioblastoma
JAK: Janus kinase
STAT3: Signal transducer and activator of transcription 3
mTOR: Mammalian target of rapamycin
FAK: Focal adhesion kinase
AXL: AXL receptor tyrosine kinase
COX2: Cyclooxygenase-2
MDSCs: Myeloid-derived suppressor cells
PERK: PKR-like endoplasmic reticulum kinase
ER: Endoplasmic reticulum
P4HA2: Prolyl 4-hydroxylase subunit alpha 2
HIF-1α: Hypoxia-inducible factor 1-alpha
5-FU: 5-fluorouracil
TPX2: Targeting protein for Xklp2
MMPs: Matrix metalloproteinases
Nrf2: Nuclear factor erythroid 2-related factor 2
sPLA2IIa: Secretory phospholipase A2 group IIa
EGFR: Epidermal growth factor receptor
MELK: Maternal embryonic leucine zipper kinase
FoxM1: Forkhead box M1
Bax: BCL2 associated X
Bcl-2: B-cell lymphoma 2
FasL: Fas ligand
IRE1α: Inositol-requiring enzyme 1 alpha
CHOP: C/EBP homologous protein
CTGF: Connective tissue growth factor
LATS1: Large tumor suppressor kinase 1
β-TrCP: Beta-transducin repeat containing E3 ubiquitin protein ligase
HERPUD1: Homocysteine inducible ER protein with ubiquitin like domain 1
SAT1: Spermine N1-acetyltransferase 1
GAS6: Growth arrest specific 6
APC: Adenomatous polyposis coli
APC: Adenomatous polyposis coli
HO-1: Heme oxygenase-1
NOX2: NADPH oxidase 2
NO: Nitric oxide
cGMP: Cyclic guanosine monophosphate
H2S: Hydrogen sulfide
KATP: ATP-sensitive potassium channel
MCP-1: Monocyte chemoattractant protein-1
PPAR-γ: Peroxisome proliferator-activated receptor gamma
TGF-β: Transforming growth factor beta
IRAK-1: Interleukin-1 receptor-associated kinase 1
CYP3A: Cytochrome P450 3A
CD163: Cluster of Differentiation 163
RTKs: Receptor tyrosine kinases
T2D: Type 2 diabetes
SPR: Surface Plasmon Resonance
ITC: Isothermal Titration Calorimetry
